# Does out of Hours Endoscopic Retrograde Cholangiopancreatography Lead to Higher Pancreatitis and Hospitalization Rates?

**DOI:** 10.1111/ans.70227

**Published:** 2025-07-02

**Authors:** Hasib Ahmadzai, Youngjun Ban, Wilson Siu, Muzhi Zhao, Andrew Thomson

**Affiliations:** ^1^ Gastroenterology & Hepatology Unit The Canberra Hospital, ACT Health Canberra Australia; ^2^ Australian National University Medical School Acton Australia; ^3^ Research School of Finance, Actuarial Studies & Statistics, Australian National University Canberra Australia

**Keywords:** ERCP, out of hours endoscopic retrograde cholangiopancreatography, post‐endoscopic retrograde cholangiopancreatography pancreatitis, prolonged hospital stay, sphincterotomy

## Abstract

**Background:**

Of endoscopic procedures, in which the skin is not breached, endoscopic retrograde cholangiopancreatography (ERCP) has by far the highest complication rate. The aim of this study was to determine if ERCP‐related morbidity is even higher if performed out of working hours.

**Methods:**

Contemporaneously collected data of 3163 patients undergoing ERCP (213 out of hours and 2929 in hours).

**Results:**

Post‐ERCP pancreatitis (PEP) occurred in 7 of 213 (3.3%) patients undergoing out of hours ERCP and 119 of 2929 (4.1%) patients undergoing in hours ERCP (*p* = 0.759). Average in hours procedure duration was 20 min, compared to 23 min in out of hours cases (*p* < 0.001). Fifty‐one patients (23.9%) in the out of hours group had a prior sphincterotomy compared with 1148 (39.2%) in hours patients (*p* < 0.001). Eighty‐seven (40.8%) out of hours patients had their procedure performed for cholangitis compared with 232 (7.9%) in hours patients (*p* < 0.005). Twenty‐five out of hours patients (11.7%) had ERCP performed for bile leak, compared with 49 (1.7%) in hours patients (*p* < 0.005). Prolonged procedure duration in the out of hours procedures remained significant with multivariate analysis.

**Conclusions:**

To the extent that there was no difference in the rate of PEP nor unplanned hospitalisation between out of hours and in‐hours cases, no evidence for increased risk of out of hours procedures was found. Out of hours ERCPs were of longer duration compared with in‐hours cases possibly due to delays related to reduced staffing and impaired logistics, rather than factors likely to increase PEP such as a higher number of cannulation attempts.

## Graphical abstract

The aim of this study was to determine if ERCP related morbidity is higher if performed out of workinghours. Contemporaneously collected data patients undergoing ERCP at an Australian hospital were analysed. No difference was found in rates of post‐ERCP pancreatitis (PEP) in patients undergoing out of hours ERCP compared with in hours ERCP (*p* = 0.759). Average procedure duration was greater for out of hours cases (*p* < 0.001); remained significant with multivariate analysis and increased procedure duration was associated with increased PEP (*p* < 0.001).

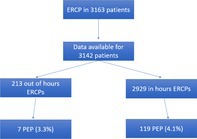

## 1 Introduction

There is long‐standing and growing interest in the morbidity and mortality of out‐of‐hours procedures and operations [1, 2]. This interest has extended to the field of endoscopy, including endoscopic retrograde cholangiopancreatography (ERCP), which has by far the highest complication rate of any endoscopic procedure in which the skin is not breached. ERCP is commonly performed by surgeons and has an important role in the management of diseases such as cholelithiasis‐related illness and pancreaticobiliary tumours, which frequently fall within the ambit of surgical practice. Post‐ERCP pancreatitis (PEP) is the most common serious procedure‐related complication, with an incidence between 3% and 6% in most trials [3]. Out‐of‐hours ERCP performed within 24–48 h of presentation is associated with reduced mortality and organ failure [4]. Out‐of‐hours ERCP, however, can be more challenging due to reduced resources, including staffing, and the burden related to the management of acutely unwell and clinically unstable patients. Patients may be acutely unwell in the out‐of‐hours setting from haemodynamic instability secondary to septic shock, coagulopathy, acute kidney injury, dehydration, or acute respiratory failure [5, 6, 7, 8]. The impact of these factors on ERCP‐related complications, including PEP, is not clear [5].

Patient related risk factors that are associated with increased risk of PEP include female gender, younger age, previous history of pancreatitis and sphincter of Oddi dysfunction. Procedural risk factors for PEP include difficult biliary cannulation, cannulation of the pancreatic duct, contrast injection into the pancreatic duct, precut sphincterotomy, biliary balloon sphincter dilatation and lack of proceduralist experience [9, 10, 11]. The possible contribution of the time of day of the procedure to clinical outcome and procedure related complications remains unclear. One retrospective analysis of 505 patients with acute biliary pancreatitis did not identify any association between timing of ERCP within 72 h with ERCP related complications or length of hospital stay [12]. On the other hand, a more recent meta‐analysis of over 7500 patients with cholangitis showed that ERCP performed within 48 h was associated with reduction in hospital mortality [4]. Data specifically related to procedure related complications were not presented in this analysis, however. An even more recent Japanese multicentre prospective cohort study of 3410 patients did not identify any difference in the rate of PEP between patients undergoing elective versus emergency ERCP [5]. The relevance of this study in other parts of the world may be limited due to the rarity of performing sphincteroplasty in the absence of prior sphincterotomy outside of Japan. We therefore aimed to investigate further for a possible out of hours effect with respect to ERCP and in particular to adjust for potential confounding patient and procedure related factors using multivariate analysis.

## 2 Methods

### 2.1 Subject Characteristics and Design

Contemporaneously collected and recorded data of all patients undergoing in hours or out of hours ERCP at The Canberra Hospital (an Australian tertiary referral centre) by a single expert operator (AT) between September 2009 and May 2022 were analysed (the “ERCP database”). Cases were categorised into in hours and out of hours based on the time of day that the procedure occurred. Procedures performed outside working hours (8 a.m. to 5 p.m. Monday to Fridays) and on public holidays were classified as “out of hours.” Irrespective of time of day, virtually all procedures were performed in the same room in the radiology department in which standard ERCP accessories were available. The cases performed out of hours were all emergencies and all were inpatients. Procedures were performed either by a single expert operator (AT) or an advanced trainee under his supervision. Of the 3163 ERCPs, full data were only available for 3142 patients, which included 2929 in hours patients and 213 out of hours patients (Table 1). Data including patient age and sex, procedure indication (as previously defined by Yuen et al. [9]), procedure duration (the time from the scope crossing the cricopharyngeus to its withdrawal from the patient), result, presence of previous sphincterotomy, whether the desired duct was deeply cannulated, whether a pancreatic stent was placed, development of PEP and unplanned hospitalisation or in the case of inpatients prolongation of hospital stay were recorded and contemporaneously compiled in an Excel database (Microsoft, Redmond, WA, USA). Following the ERCP procedure, patients were followed‐up proactively with phone calls by the endoscopist on the evening of the procedure and the following day. For inpatients, the endoscopist reviewed the patients in person on the ward or contacted the attending nursing and/or junior medical staff both on the evening of the procedure and the next day. For patients with unplanned hospital stay, their clinical course was monitored. PEP was defined as significant persisting new post procedure abdominal pain with a serum lipase elevation at least five times the upper limit of normal. Prolonged hospital stay was defined as any unplanned hospital admission due to an ERCP related complication in the case of outpatients, or prolonged hospital admission related to such complications for inpatients [13].

**TABLE 1:** Subject characteristics.

	Elective ERCP group	Emergency ERCP group	*p*
	(*n* = 2929)	(*n* = 213)	
Age, median (range)	67 (52–79)	70 (53–81)	0.174
Gender, *n* (%)			
Female	1688 (57.6)	99 (46.5)	**0.002**
Male	1241 (42.4)	114 (53.5)	
Procedure duration (mins) median (IQR)	20 (13–30)	23 (15–40)	**< 0.001**.
Outpatient versus inpatient, *n* (%)			
Inpatient	1269 (43.3)	213 (100%)	**< 0.001**
Outpatient	1660 (56.7)	—	
Procedure indication, *n* (%)			
Ascending cholangitis	233 (7.9)	87 (40.8)	**< 0.001**
Acute biliary pancreatitis	243 (8.3)	20 (9.4)	0.607
Bile leak	49 (1.7)	25 (11.7)	**< 0.0001**
IOC	246 (8.4)	11 (5.2)	0.119
Secondary indication^a^	1267 (43.3)	58 (27.2)	**< 0.0001**
C/ROS B	666 (22.7)	2 (0.9)	**< 0.0001**
C/ROS M	126 (4.3)	2 (0.9)	0.011
Other	99 (3.4)	8 (3.8)	0.696
Deep access to bile duct, *n* (%)	2755 (94.1)	197 (92.5)	0.353
Prior sphincterotomy, *n* (%)	1148 (39.2)	51 (23.9)	**< 0.001**
Performance of sphincterotomy, *n* (%)	1770 (60.4)	160 (75.1)	**< 0.001**
Pancreatic stent, *n* (%)	328 (11.2)	27 (12.7)	0.511

*Note:* For continuous variables a *t*‐test was used, otherwise, a *χ*‐squared test was used to calculate *p*‐values, Ref. [9]. The bold values refer to significant *p*‐values in Table 1.

Abbreviations: C/ROS B, change or removal of biliary stent for benign disease; C/ROS M, change or removal of biliary stent for malignant disease; IOC, positive intra‐operative cholangiogram.

^a^ Secondary indications also include two or more of: biliary pain, imaging evidence of biliary or pancreatic ductal abnormality and deranged liver function tests.

The ERCP procedures were mainly performed under conscious sedation administered by a specialist anaesthetist with intravenous injection of propofol together with midazolam or fentanyl or both of these drugs. The vast majority were performed in the same X‐ray room in the radiology department. Most ERCPs were performed without resort to endotracheal intubation. For standard biliary cannulation, the wire‐guided cannulation technique was used. In cases of difficult cannulation, a variety of other techniques including the double wire technique and the use of precut sphincterotomy were performed. For prophylaxis against PEP, rectally administered non‐steroidal anti‐inflammatory drugs including diclofenac were used as well as intravenous fluid hydration after evidence supporting their use came to light. The data were cross‐referenced with the hospital medical records for cases of missing data. The study was approved by the ACT Health Human Research Ethics Committee.

### 2.2 Statistical Analyses

Data were expressed as mean and standard deviation. The incidence and characteristics of PEP between out of hours and in hours ERCP were compared using both univariate and multivariate logistic regression models. The two‐sample *t*‐test was used for continuous variables and *χ* ^2^ test to compare categorical variables. Associations are presented using odds ratios (ORs) with 95% confidence intervals (CIs). When analysing the effects of covariates on binary variables the likelihood ratio test was used. A *p*‐value of ≤ 0.05 was considered statistically significant. All statistical analyses were performed using R (version 4.3.2, University of Auckland, Auckland, New Zealand).

## 3 Results

A summary of the subject characteristics from our cohort of patients is listed in Table 1. In the overall cohort of patients, there was a significant difference in the gender of patients undergoing in hours (57.6% female) compared with out of hours ERCP (46.5% female) (*p* = 0.002). There was also a significant difference between procedure duration, with out of hours procedures having a median duration of 23 min compared with 20 min in hours cases (*p* < 0.001). All out of hours cases were performed as inpatient procedures, compared with 43.3% of in hours ERCP cases, which were inpatients (*p* < 0.001). A greater proportion of in hours ERCP cases (39.2%) had a previous sphincterotomy performed compared with out of hours cases (23.9%) (*p* < 0.001), whereas, as expected, an initial sphincterotomy was performed in a greater proportion of patients undergoing out of hours ERCP (*p* < 0.001).

PEP occurred in 7 of 213 patients (3.3%) undergoing out of hours ERCP, and 119 of 2929 patients (4.1%) undergoing in hours ERCP (*p* = 0.577). Unplanned hospitalization or extension of hospital stay including cases of PEP occurred in 278 of 2929 (5.6%) of the out of hours group compared to 12 of 213 (9.5%) of the out of hours group (*p* = 0.6). Of the unplanned hospitalization cases in the out of hours group none occurred due to post sphincterotomy bleeding. Comparing procedural indications, 87 (40.8%) patients in the out of hours group had their procedure performed for cholangitis compared with 232 patients (7.9%) in the in hours group (*p* < 0.005). There were 25 patients (11.7%) who had ERCP performed for bile leak in the out of hours group; compared with 49 patients (1.7%) in the in hours group (*p* < 0.005).

With respect to the entire cohort, there were univariate associations between a range of parameters and PEP (see Table 2). However, when multivariate analysis was performed, factors that were associated with development of PEP only included longer procedure duration (*p* < 0.001), outpatient procedures (*p* = 0.004), ERCP in acute pancreatitis (*p* < 0.001) and pancreatic stent insertion (*p* = 0.007). The risk of PEP was lower with biliary stent change for both benign (*p* < 0.001) and malignant disease (*p* = 0.033).

**TABLE 2:** Predictive risk factors for PEP using univariate and multivariate analysis.

	Non‐PEP, *n* (%) (*n* = 3016)	PEP, *n* (%) (*n* = 126)	Univariate logistic regression		Multivariate logistic regression	
			OR (95% CI)	*p*	OR (95% CI)	*p*
In hours group, *n* (%)	2810 (93.2)	119 (94.4)				
Out of hours group, *n* (%)	206 (6.8)	7 (5.6)				
Out of hours versus In hours			0.86 (0.34–1.62)	0.578		
Age, median (IQR)	68 (52–79)	64 (52–75)	0.99 (0.98–1)	0.048		
Gender, *n* (%)				0.413		
Female	1710 (56.7)	77 (61.1)				
Male	1306 (43.3)	49 (38.9)	0.83 (0.58–1.2)	0.328		
Duration, median (IQR)	20 (13–30)	27 (20–39)	**1.02 (1.01–1.03)**	**< 0.001**	**1.02 (1.01–1.03)**	**< 0.001**
Inpatient	1424 (47.2)	56 (44.4)				
Outpatient	1592 (52.8)	70 (55.6)				
Inpatient versus outpatient			1.12 (0.78–1.6)	0.542	**1.77 (1.2–2.64)**	**0.004**
Prior sphincterotomy, *n* (%)	1166 (38.7)	33 (26.2)	**0.56 (0.37–0.83)**	**0.005**		
Indication, *n* (%)						
Acute pancreatitis	248 (8.2)	14 (11.1)		**< 0.001**		**< 0.001**
Bile leak	70 (2.3)	4 (3.2)	1.01 (0.28–2.93)	0.983	1.02 (0.28–3.02)	0.978
Stent change (benign disease)	658 (21.8)	9 (7.1)	0.24 (0.1–0.56)	**0.001**	0.2 (0.08–0.47)	**< 0.001**
Stent change (malignant)	128 (4.2)	1 (0.8)	0.14 (0.01–0.7)	0.057	0.11 (0.01–0.56)	**0.033**
Cholangitis	311 (10.3)	8 (6.3)	0.46 (0.18–1.08)	0.082	0.52 (0.2–1.26)	0.155
Positive IOC	245 (8.1)	14 (11.1)	1.01 (0.47–2.18)	0.975	0.87 (0.4–1.9)	0.724
PIL	1253 (41.5)	71 (56.3)	1 (0.57–1.88)	0.99	0.9 (0.51–1.71)	0.722
Performance of sphincterotomy, *n* (%)	1833 (60.8)	97 (77)	**2.16 (1.44–3.35)**	**< 0.001**		
Pancreatic stent, *n* (%)	325 (10.8)	30 (23.8)	**2.59 (1.67–3.91)**	**< 0.001**	1.83 (1.16–2.81)	**0.007**
Unplanned admission/prolonged stay	165 (5.5)	125 (99.2)	**2159 (479–38 131)**	**< 0.001**		

*Note:* The bold values in Table 2 indicate significant odds ratios and *p*‐values.

## 4 Discussion

To the extent that complication rates including PEP were similar between out of hours and in hours ERCP, no evidence of increased risk of out of hours procedures was found in our study. Our results were similar to the recent Japanese study which demonstrated no significant difference in the rate of PEP between patients undergoing emergency and those having elective ERCP procedures [5]. Interestingly, in our series, patients in the out of hours cohort had a longer average procedure duration. However, despite this there was no difference in the rate of PEP nor unplanned hospitalization/prolongation of hospital stay between the out of hours and in hours groups. Notwithstanding the above, our group and others have previously shown that a longer ERCP procedure time is in fact linked to ERCP related complications [10, 14]. This suggests that the prolongation of the ERCP in out of hours cases may relate to procedure lengthening factors that are not associated with an increased risk of complications, such as multiple unsuccessful attempts at biliary access. There is only one endoscopy nurse present during out of hours ERCPs in comparison to in hours procedures during which at least two nurses are generally present. Response time to endoscopist requests is thus slower particularly if an extra item of equipment needs to be obtained from another area of the hospital during the procedure. In addition, on call radiographers are deployed for out of hours ERCPs and they may not be as familiar with the image intensifier equipment as radiographers designated to in hours ERCP lists. Another factor that may have mitigated the risk of complications in the out of hours group is that very few of the out of hours ERCPs were performed between midnight and 06:00 a.m.—a period in which attending endoscopists may be at particular risk of fatigue related impairment in performance. In addition, the fact that the out of hours ERCPs were performed in the same room with the same, albeit reduced, nursing staff with the same proceduralist may have contributed to a lower complication rate. It is acknowledged that many of the staffing and logistics issues were present if emergency ERCPs were performed in hours at a time other than that of a regular ERCP list. However, this occurred rarely and was not systematically recorded.

Other potential sources of significant difference between the two groups include possible differing levels of use of standard PEP prevention strategies such as NSAID use and pre‐procedure IV fluid administration. After out‐of‐hours cases, it has been the authors' observation that NSAIDs are less likely to be given as these patients are recovered in a different area from the recovery area used during working hours and sometimes the NSAIDs may thus not be available. In addition, intensive care unit staff can be less enthusiastic about NSAID use if patients are exhibiting systemic inflammatory response syndrome with potential or actual renal impairment. Similarly, a fixed aggressive fluid hydration strategy was not used in many of the out‐of‐hours cases as additional fluids had already been given in many instances for hemodynamic instability and dehydration. There was also a tendency in the out‐of‐hours group for less risky interventions such as sphincteroplasty to be performed. This may have contributed to a lower risk of post‐ERCP pancreatitis and other complications in this group. The extent to which these factors altered the PEP rate between the two groups remains a matter for conjecture as these parameters were not recorded systematically in our series. On the other hand, coagulopathy related to sepsis would be expected to increase the risk of post‐procedure bleeding in the out‐of‐hours group. In this vein, in some cases, there may have been inadequate time to wait for anticoagulation medication and, in particular, anti‐platelet medication to wear off. In our series, however, there was no significant bleeding in the out‐of‐hours cases. This may have been due at least in part to rigorous attention to reversal of anticoagulation in the emergency phase of management and the performance of shorter sphincterotomies in those at risk of bleeding.

In terms of the overall cohort, there were several factors associated with the development of PEP (including procedure duration and pancreatic stent placement) but when these factors were adjusted for using multivariant analysis, no difference between the two groups was evident. There was no difference in the rate of pancreatic duct stent insertion between in hours and out of hours cases. Although pancreatic stent insertion lowers the risk of developing PEP [15, 16, 17], it conferred a greater risk in our study, as pancreatic stents were placed almost exclusively when the pancreatic duct was inadvertently entered when biliary ductal access was desired—an event which in and of itself increases the risk of PEP. Thus, the rate of PEP would almost certainly have been even higher if the pancreatic duct stents had not been placed.

One limitation of our study was that some of these additional factors, including those associated with PEP risk, such as the number of failed biliary cannulation attempts, frequency of contrast injection into the pancreatic duct, and performance of balloon sphincteroplasty, were not analysed [5, 10, 18]. The authors also appreciate the other limitations in this study, including its relatively small size and its coming from a single centre. The data analysed were retrospective, notwithstanding their contemporaneous collection. The data collection in this study, however, was more assiduous than is the case for other studies, as the endoscopist, rather than largely relying on information from third parties and medical records, followed the patients on an individual basis both on the evening of the procedure as well as the following day to ensure complications were not missed. In addition, the involvement of an experienced practitioner of ERCP in all out‐of‐hours procedures may have prevented morbidity related to out‐of‐hours ERCPs from occurring. To the extent that experienced ERCP practitioners are few in number, this interpretation of the findings implies that it may be in the interests of patient care if such practitioners be available out of hours to do ERCPs or that early referral to other facilities or even other cities be accepted practice with respect to the management of patients requiring out‐of‐hours ERCPs. Transferring patients to high‐volume centres with established pathways for the performance of out‐of‐hours ERCPs is advised. These sorts of manpower and logistics issues are not unique to pancreatobiliary surgery in that there has been ever‐increasing superspecialisation of surgical practice more generally in the first quarter of this century.

In conclusion, notwithstanding the procedure time of out of hours ERCPs being significantly longer than in hours ones, no significant differences were identified between the rate of PEP in out of hours and in hours ERCPs, with a PEP rate of about 4% in both groups.

## Author Contributions

**Hasib Ahmadzai:** conceptualization, investigation, writing original draft. **Youngjun Ban:** conceptualization, investigation. **Wilson Siu:** investigation. **Muzhi Zhao:** data curation, formal analysis. **Andrew Thomson:** conceptualization, investigation, supervision, writing original draft, writing – review and editing.

## Conflicts of Interest

The authors declare no conflicts of interest.
